# Account of Diseases in an Eastern District of London, from May 20 to June 20, 1804

**Published:** 1804-07-01

**Authors:** 


### *Account of Diseases in an Eastern District of London, from May 20 to June 20, 1804.*

| | |
|---|---|
| **ACUTE DISEASES.** | |
| Febris Intermittens - - | 1 |
| Pleuritis - - - - - - | 2 |
| Rheumatismus Acutus - | 4 |
| **CHRONIC DISEASES.** | |
| Dyspnœa - - - - - - | 8 |
| Tussis cum Dyspnœa - | 14 |
| Hæmoptysis - - - - - | 3 |
| Pleurodyne - - - - - | 2 |
| Epilepsia - - - - - - | 1 |
| Cephalalgia - - - - - | 4 |
| Convulsio - - - - - - | 1 |
| Paralysis - - - - - - | 3 |
| Syncope - - - - - - | 2 |
| Ascites - - - - - - - | 4 |
| Hæmorrhois - - - - - | 3 |
| Chlorosis - - - - - - | 6 |
| Scrophula - - - - - - | 5 |
| Rheumatismus Chronicus | 10 |
| **PUERPERAL DISEASES.** | |
| Enteritis - - - - - - | 2 |
| Menorrhagia Lochialis - | 5 |
| Ephemera - - - - - - | 6 |
| **INFANTILE DISEASES.** | |
| Pertussis - - - - - - | 6 |
| Scrophula - - - - - - | 2 |
| Herpes - - - - - - - | 2 |
| Tinea - - - - - - - | 1 |
| Vermes - - - - - - - | 2 |

